# Magnetic and Electric Energy Harvesting Technologies in Power Grids: A Review

**DOI:** 10.3390/s20051496

**Published:** 2020-03-09

**Authors:** Feng Yang, Lin Du, Huizong Yu, Peilin Huang

**Affiliations:** 1 College of Engineering and Technology, Southwest University, Chongqing 400716, China; yangfengswu@swu.edu.cn; 2 State Key Laboratory of Power Transmission Equipment and System Security and New Technology, Chongqing University, Chongqing 400044, China; 20143708@cqu.edu.cn (H.Y.); 201911131075@cqu.edu.cn (P.H.)

**Keywords:** energy harvesting, electric field energy, magnetic field energy, power grid

## Abstract

With the development of intelligent modern power systems, real-time sensing and monitoring of system operating conditions have become one of the enabling technologies. Due to their flexibility, robustness and broad serviceable scope, wireless sensor networks have become a promising candidate for achieving the condition monitoring in a power grid. In order to solve the problematic power supplies of the sensors, energy harvesting (EH) technology has attracted increasing research interest. The motivation of this paper is to investigate the profiles of harnessing the electric and magnetic fields and facilitate the further application of energy scavenging techniques in the context of power systems. In this paper, the fundamentals, current status, challenges, and future prospects of the two most applicable EH methods in the grid—magnetic field energy harvesting (MEH) and electric field energy harvesting (EEH) are reviewed. The characteristics of the magnetic field and electric field under typical scenarios in power systems is analyzed first. Then the MEH and EEH are classified and reviewed respectively according to the structural difference of energy harvesters, which have been further evaluated based on the comparison of advantages and disadvantages for the future development trend.

## 1. Introduction

Emerging technologies in power systems such as smart grid (SG) and ubiquitous power Internet of things (UPIOT) have attracted considerable interest recently [1,2,3]. As an underlying process, the perception of the operational condition in the grid and measurement of the grid parameters plays an important role in achieving system intelligence. Since the large-scale networks are expected to be mostly manipulated “intelligently”, massive and real-time information needs to be collected, in particular the parameters associated with critical power equipment, which enable the smart-grid to respond to changing conditions proactively. Such parameters may be associated with the operational conditions (e.g., voltage, current), or the meteorological factors at the deployed site (e.g., temperature, humidity) [4,5].

Technically, wireless sensor networks (WSNs) are most promising to implement the measurement tasks [6,7]. However, in many cases, due to the limitations of field conditions, traditional solutions have been disabled to effectively provide power supplies for monitoring devices, which has become one of the bottlenecks that restrict the deployment of WSNs. For facilities located in remote areas, such as overhead transmission lines, it is usually difficult to find the available low-voltage power supply nearby. Even if available, for the sake of insulation safety, some sensor nodes at the high potential in substations cannot be powered by cabling. On the other hand, it is often impractical to operate these systems on a fixed energy source like a battery owing to the difficulty in replacing the battery. For instance, a 1 cm^3^ primary lithium battery has a typical energy storage capacity of 2800 J. This can potentially supply an average electrical load of 100 W for close to a year but is insufficient for systems where battery replacement is not an easy option [8]. Besides, batteries encounter long-term reliability and efficiency problems in harsh environments. For example, in outdoor applications like under the sunlight, the environment temperature may violate the operational range of Li-ion batteries, which may cause overheating or even explosion and intensify the self-discharge process. Therefore, it is indeed an underlying paradox that although in the vicinity of large energy flow under the environment of the power grid, there are no easy ways in a high-voltage facility to directly wire a power source by connecting to the grid or using the so-called on-grid power.

In recent years, with the continuous reduction of power consumption of sensors, microprocessors, and radio-frequency (RF) modules, as well as the breakthrough of ultra-low energy management techniques, the feasibility of a micro energy-autonomous system has been improved, which facilitates the further development of energy harvesting (EH) technology. EH means scavenging and transforming various forms of stray energy from the environment into electricity [9,10]. There are several EH methods that are research-focused, including solar, thermal, electromagnetic and vibrational EH technologies. For example, some novel vibration-based energy harvesting technologies have come forth using piezoelectric materials or triboelectric phenomena. M. Peddigari et al. designed a piezoelectric energy harvester (PEH) with a cantilever structure [11]. The device using hard and soft piezoelectric materials exhibited the maximum output power of 3.18 mW and 2.53 mW, respectively, at the resonant condition. A microcube-based piezo composite with a metal–insulator–metal structure was fabricated, which produced a maximum instantaneous power density of 50 μW cm^−3^ at a load resistance of 160 MΩ [12]. Analogously, a highly enhanced piezo composite energy harvester with biomimetic porifera skeletal structure obtained an output power density of 0.2 μW cm^−2^ [13].

Until now, EH has been widely used to provide power supplies for sensor nodes in structural health monitoring, medical implantable devices, military monitoring devices, remote weather stations or large-scale renewable energy generation [14]. Therein, the most prominent application is to energize wireless sensors [15,16]. For instance, the magneto-mechano-triboelectric generator was designed using piezoelectric and magnetostrictive materials, which convert magnetic noise to useful electric energy applying for low-power consuming electronics or wireless sensor networks [17,18,19]. RF energy could be converted to electric energy through a circuit mainly consisting of an antenna, impedance matching circuit, rectifier, voltage multiplier and energy storage for energizing low-power wireless devices [20].

From the perspective of available resources, there are many location-dependent sources of harvestable energy in the context of a power grid, such as solar, electric field, magnetic field, thermoelectric and vibration-based energy. Among them, the power grid is awash in the power frequency magnetic field (H-field) and electric field (E-field), which are excited by the energized high voltage (HV) conductors carrying high currents in power plants or substations. Consequently, the amount of potential H-field and E-field energy is promising and can be the most achievable. Until now, a considerable body of literature has reviewed harvesting natural sources, like solar and wind energy, or recovering waste energy, like thermal and vibrational energy. However, under the very specific context of power grids, H-field and E-field energy should be more intensively studied on behalf of field applications, as their available amount is significantly higher than the other energy forms, while any systematic review on this part is infrequently reported. Therefore, in view of their practicability as well as the fact that the other EH methodologies have scarcely been actually deployed in the power grid yet, the present contribution will specially review the magnetic and electric field energy harvesting technologies. The authors clarify that the so-called magnetic and electric energy harvesting technologies hereinafter are only limited to the capacitive and inductive approaches, while the other scavenging mechanisms will not be discussed. To this end, the rest of the paper is organized in the following manner. Section 2 investigates the profiles of harvest energies in the context of a power grid so as to signify its feasibilities. Section 3 and Section 4 review the magnetic and electric energy harvesting technologies, respectively, in accordance with the structural difference of energy harvesters, in which the fundamentals, current status, challenges, and future prospects are analyzed.

## 2. Profiles of Potential Harvestable Energy in a Power Grid

**Figure 1:**
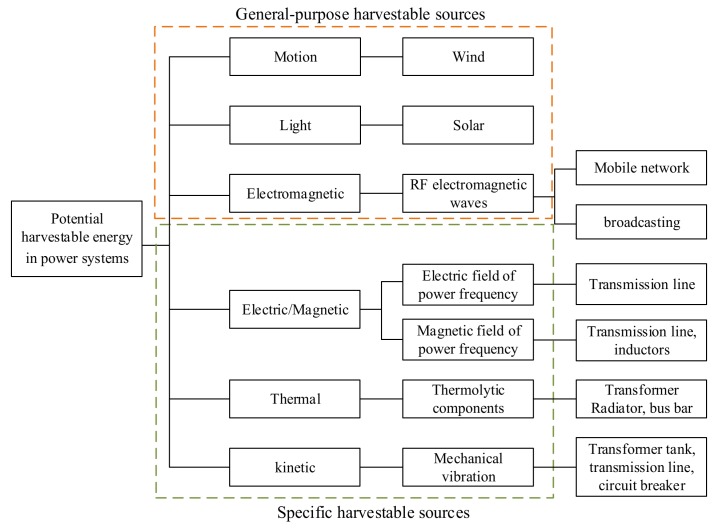
Profiles of harvestable energy sources in the context of power systems.

Different from other EH occasions, as a power system is the hub of energy conversion, there will be indisputably abundant energy reserves in the vicinity of the grid, whether it is the product of the power grid—electric energy, or the dissipation energy accompanying the generation, transformation and transmission of electricity, like waste heat and kinetics energy. In this sense, there are inherently more possibilities and feasibility to achieve EH in a power grid. In addition, lots of power infrastructures are distributed outdoors, such as overhead transmission lines and substations. Therefore, some general-purpose EH approaches can also be employed, such as solar and wind energy. Similar to other industrial application scenarios, except for scavenging energy from H-field and E-field, all the other feasible EH methods in the power grid can include photovoltaic, thermoelectric, mechanical and electromagnetic (RF) energy forms, despite some of them not being practically deployed. Based on the pertinence differences orienting power systems, the above EH approaches are divided into two categories: general and special EH methods, as shown in Figure 1. As a farmer must familiarize and prepare the land to harvest a predictable and useful crop, the potentials of an environment for harvesting enough energy to produce a useful application must be clearly understood first. The profiles of ambient energy distribution in power grids are analyzed in the following.

The power equipment in operation continuously dissipates waste heat into the ambient, which may result in hot spots with significantly higher temperatures on the casings or any parts of the equipment, which actually forms an environmental heat source. This phenomenon can be easily found on a transformer casing, bus bar, etc. By reasonably designing the heat collector and heat sink structure, the temperature difference can be generated between both ends of thermoelectric modules so that heat can be transformed into electricity. For instance, in reference [21], the temperature profiles of a 63 MVA oil-immersed and air-cooled transformer were measured. The measured data showed that the highest temperature point outside the transformer was located in the middle of the oil tank and at the top of the radiator, which was between 333 K and 353 K. If the room temperature is considered to be 25 °C, the maximum temperature difference can reach 55 °C.

Assuming that the commercial thermoelectric generator (TEG) module TEHP1-1263-1.5 is used, it is composed of 126 pairs of semiconductor couple arms. According to Formulas (1)–(3) in the literature [22], under the above temperature profiles of the transformer and the matched loading condition, the maximum power of the module can be calculated to be 475 mW.

(1) $$P_{e\max}=\frac{N\alpha^{2}\Delta T^{2}A}{2\rho l}$$

*α*_P_ = −*α*_N_ = (22224.0 + 930.6*T*_m_ − 0.9905*T*_m_^2^) × 10^−9^ V/K (2)

*ρ*_P_ = *ρ*_N_ = (5112.0 + 163.4*T*_m_ + 0.6279*T*_m_^2^) × 10^−10^ Ω/m (3)

The kinetic energy present in vibrations is another potential source, which can be readily found in transportations, buildings, industrial machinery and so forth. For the transformer in operation, the magnetostriction of silicon steel sheet, the electromagnetic force caused by leakage current between silicon steel sheet joint and lamination, the magnetic leakage caused by load current in winding will all cause vibration of the transformer tank. For instance, data from a recent study shows that the vibration level of an in-service 132/66 kV, 40 MVA transformer is above 1.0 m/s^2^ for most of the time. A Perpetuum PMG (Southampton, England) 17 vibration energy harvester could produce 4.5 mW under these conditions [23]. Except for the vibration of transformers, the overhead transmission line will vibrate due to the action of wind load. Besides, the operating of circuit breakers in substations will also cause a large amplitude of transient vibration.

For the outdoor scenarios of power systems, traditional and general harvesting approaches can be used, such as solar energy, wind energy, and electromagnetic (RF) EH methods, the deployment considerations of which are totally identical to those under other industrial scenarios. Because of this universality, a considerable body of literature has reported on these general EH methods. However, in closed situations such as indoor distribution rooms and cable ducts, the general EH methods become inapplicable. Under this circumstance, some specific EH methods should be investigated, e.g., electric field energy harvesting (EEH) and magnetic field energy harvesting (MEH). As alternative EH solutions, thereby, EEH and MEH can provide significantly more amount of available power.

It is clarified first that the so-called EEH and MEH refer to scavenged energy from H-field and E-field of power frequency, which are different from RF energy harvesting. EEH and MEH are specially reviewed here as lots of components carrying high currents are present in the power grid, such as the bus bars in switchgear. Therefore, an intensive magnetic field of power frequency is bound to be induced surrounding the conductors. H-field energy can be scavenged through electromagnetic induction. On the other hand, intensive E-field is present in the vicinity of high potential conductors.

Strictly, EEH and MEH are “stealing” rather than harvesting or recovering waste energy, because the very essence of electricity lies in the electromagnetic field itself. Under power frequency, the interaction between E-field and H-field can be disregarded, which are only generated by charge and current respectively, and thus quasi-static E-field and H-field can be decoupled, leading the two kinds of energy can be scavenged separately. In a typically designed substation, when 35 kV three-phase bus bars carry standard loading currents of 1039 A, the H-field on the surface of the bus bar can reach 100 mT [4]. In literature [23], the authors experimentally showed that maximum energy of 148 μJ/m^3^ can be harvested from a 400 kV substation having a maximum E-field strength of 5.8 kV/m.

Based on the above analysis, on the one hand, EH actually makes full use of the abundant resources of electromagnetic energy in the grid. On the other hand, it can also solve the problematic power supplies of wireless sensor networks. In this sense, EH is quite a promising solution and therefore attracts lots of research interest.

## 3. Magnetic Energy Harvesting Technologies

### 3.1. Fundamentals of MEH

The conductors in power systems such as overhead lines, cables, and bus bars usually carry large power frequency load current, and power frequency H-field is present. Due to the existence of the alternating field, the induced voltage can then be generated inversely through the electromagnetic induction using a coil. This kind of harvester is essentially an inductor, so MEH is also called inductive energy harvesting. The induced alternating voltages must be adapted using an energy management module so that stable low-voltage direct current (DC) power can be obtained and fed to the sensor loads. Conventionally, the energy management module integrates overvoltage protection, rectification, wave filtering, DC-DC conversion, and other submodules. At present, the correlated research mainly focuses on the improvement of the core material and the power management circuit.

**Figure 2:**
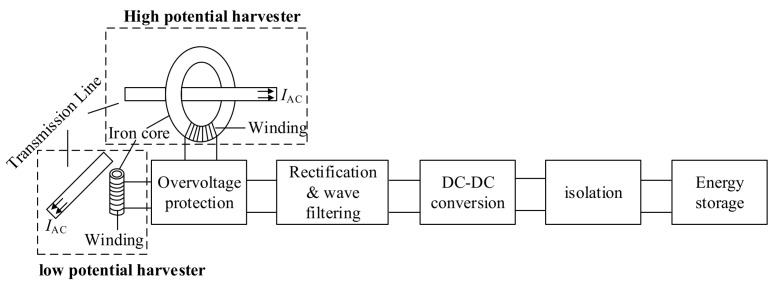
Schematic diagram of a magnetic energy harvesting system.

According to different application modes of the front-end harvester, the MEH design can be divided into two categories: high potential and low potential MEH methodologies as shown in Figure 2 below. Such classification is based on whether the overall potential of the MEH system is consistent with the target HV conductor. Meanwhile, as the difference of installation pattern under the two potential modes, the energy harvesting coil also varies in its structure. In the following, the progress of the MEH investigation will be reviewed separately in accordance with the two MEH modes.

### 3.2. Low-Potential MEH

The second type is the low-potential or stand-alone MEH structure. The stand-alone energy harvester is an independent combination of iron core and a coil that is wired on the core, the entire set of which is away from the HV conductors. It can be deployed anywhere for MEH as long as there is H-field distribution [24,25]. Conventionally, the harvester is in low potential as it will not be installed on the HV end; therefore, it is also known as the low potential MEH method. The advantage of the low potential MEH is higher flexibility, which can be applied to more scenarios. Theoretically, low potential MEH can be used for the sensors or systems to monitor any interested operational parameters as long as the magnetic field is present and the provided energy of MEH is enough. Besides, the coil in a low-potential MEH device can be miniaturized, which can also be compatible with the fabrication of micro-electro-mechanical system (MEMS).

In a low-potential MEH device, since the stray H-field is commonly weak, the most critical considerations are focused on designing the coil so as to improve the efficiency from the front end. In the literature [26], the authors proposed a novel helical core to intensify the flux density and 400 turns of wire can have a power density of 2.1 µW/cm^3^ when placed in a magnetic flux density of 7 µTrms. In literature [27], W. Jiang, et al. proposed a non-intrusive power supply for the sensor load by harvesting the near field magnetic energy from the supply cables and more than 30 mW from the power cable with 10 kHz-7A RMS ripple component. S. W. Wright et al. proposed an inductive method for harvesting energy from current-carrying structures. A rectangular ferrite core was used which can provide a × 4 power density improvement. The power density of 36 μW/g (103 μW/cm^3^) was obtained from a spatially distributed 30 A current at 300 Hz and a 1:7 funnel core demonstrate [28]. C. Cepnik and U. Wallrabe studied a flat micro energy harvester with a volume of 0.9 cm^3^ and a height of 3 mm, in which a serpentine coil having a single winding was used, and measured normalized power of 8.3 μW_avg_/(ms^−2^ cm^3^)^2^ was obtained [29].

### 3.3. High-Potential MEH

Different from low-potential MEH, the coil structure of high-potential MEH is ring-type [30,31,32]. In this way, the ring-shaped closed iron core is clamped on the section of a current-carrying conductor. The radial H-field generated by the current can be scavenged in the iron core from the secondary induced voltage. Due to the high permeability of the iron core, the energy density of high-potential MEH is comparatively large. In order to ensure the insulation safety, the harvester and also the sensor load must maintain the same high potential as that of the HV conductor. Therefore, this kind of MEH configuration is also called high potential magnetic field energy harvesting and can only be deployed for the devices with transmission lines monitoring purposes. The most significant advantage of high-potential MEH is that the scavenged power can be plentiful, which can support the application with power consumption in the order of several watts, such as video monitoring the condition of transmission lines. Besides, the condition of produced power is stable as long as the line is energized, which is always meaningful as the condition monitoring is only needed when the line is in service.

According to the different patterns of controlling the energy delivered from the primary side to the loading side, high-potential MEH methods can be classified into three types: improved Rogowski coil, current-limiting current transformer (CT) and compensative CT. For the improved Rogowski coil, the power transmission is controlled by changing the structure, material and winding turns of the coil. The original Rogowski coil is coreless with the permeability of vacuum, which features good linear characteristics and is applicable to obtain a proportional voltage signal for large current measurement rather than scavenging energy from the currents. Therefore, some efforts are made to improve the Rogowski coil mainly for drawing out the H-field energy. Representative work has been reported in the literature [33,34,35]. For the coil with given structures, the coupled energy is dominated by the H-field around the line or the line current. However, the current of the transmission lines is affected by the grid load, which usually fluctuates periodically within a range. Therefore, the adaptability of the MEH system regarding the current ranges needs to be enhanced, for which the additional current limitation module is employed. Representative work has been reported in the literature [35,36,37]. Another approach is to add a compensation coil to the primary side to control the delivered power. When the primary current increases, the secondary induced voltage also does, and the compensation coil is connected to partially counteract the H-field generated from the primary current, so as to reduce the energy transmitted to the secondary side; when the primary current decreases, the compensation coil is disconnected [38,39,40].

However, aside from the above technical problems, the high-potential MEH mode has the limitations that it may be inapplicable if it is not allowed to de-energize the high-voltage line to implement the harvester installation or there is not enough space left between the three-phase conductors to install the harvester, or the sensor load is located with low potential and any electrical connection between the sensor and the harvester may compromise the insulation [25]. In summary, the H-field inductive energy harvesting features the advantages of compact structures, simple insulation packages, and satisfactory safety. However, as the induced voltage is positively correlated with the current in HV lines, its operation will definitely be significantly affected by the grid current. As the dynamic range of the loading current is usually wide, the following two technical difficulties exist: when the primary current is lower than a few amperes, the MEH harvester cannot scavenge enough power, resulting in a “dead zone” of the harvesting system; when the primary current increases to the orders of kA, high-voltage pulse may arise in the secondary side of the coil, which may cause interference and damage to the devices on the loading side.

## 4. Electric Energy Harvesting

### 4.1. Fundamentals of EEH

The HV terminals (such as transmission lines, cables, and bus bars) in a power system are not only carrying currents but also maintain a high potential at the same time. Similar to H-field, E-field of power frequency is present in the vicinity of HV conductors. The time-varying E-field of power frequency is a quasi-static field, and the induced E-field is far less than the Coulomb field. The Coulomb E-field is the main component; therefore, EEH can be considered to harvest the electrostatic field from energized transmission lines or power lines. In the electrostatic field, there is usually a potential difference in different positions along the direction of E-field. When two electrodes are connected to the load, there will be current flowing through the load due to potential difference, so that the E-field energy is extracted. The advantage of EEH is that the induced voltage can almost remain unchanged as the under normal operating conditions, the voltage of the transmission line always remains within a small allowable range and does not fluctuate significantly, which is different from the loading current that varies periodically with the peak and trough loading conditions.

**Figure 3:**
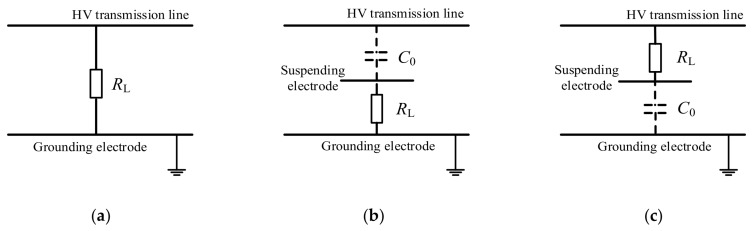
Schematic diagram of energy harvesting under electrostatic field; (**a**) direct-mode EEH; (**b**) low-potential EEH; (**c**) high-potential EEH.

Compared with MEH, there are fewer investigations on EEH, which mainly focuses on the environment with intensive E-field present, like substations or the overhead transmission lines. As the EEH harvester is essentially capacitors, EEH is also called capacitive energy harvesting. Using the principle of capacitive voltage-division under alternating E-field, the available AC low voltage can be obtained at the loading terminal, which can then be adapted into DC voltage through a power management module. According to different connection modes and the loading position on the capacitor arm, EEH can be divided into the following three types, as shown in Figure 3, where *R*_L_ in the figure represents the load. The electrodes at both ends of *R*_L_ can be high-voltage wires, grounding electrodes or spatial electrodes (as long as their potential is different). *C*_0_ is the spatial capacitance between one spatial electrode and the other (with air as the dielectric). In the following, the progress of EEH research will be reviewed in accordance with the above classifications.

### 4.2. Direct-Mode EEH

In the direct connection mode, the whole EEH system directly withstands the full operating voltage of the target HV terminal. As a load of the EEH system (monitoring sensors or devices, etc.) usually specifies low-voltage (5V DC, or 12V DC, etc.), an off-the-shelf voltage divider is necessary within the EEH in this direct mode.

X. Zhang et al. connected the high-voltage capacitor voltage divider between the high-voltage bus and the ground. After the energy obtained from the high-voltage bus is rectified, filtered and stabilized, it supplies power to the high-voltage side circuit [41]. Q. Li et al. used a special capacitor and voltage transformer in series to realize the partial voltage to obtain the electric energy directly from the 110 kV high-voltage conductor, and the output power can reach 100 W [42]. The disadvantage of direct-mode EEH is that the EEH equipment is directly connected to the high-voltage conductor, which requires satisfactory insulation performance of the voltage divider; otherwise, deployment of EEH may endanger the operation safety of the transmission line due to insulation failure.

### 4.3. High-Potential EEH

In both the following high- and low-potential EEH, the energy harvesting module will not be directly connected with the target HV conductor. Instead, E-field energy is drawn out through capacitive induction. Comparatively, such indirect and capacitive EEH methods can be more reliable and lots of relevant investigations have been reported. Zhao et al. proposed to collect the stray static electric field energy around the power line by using the cylindrical electrode sleeve to form the high-voltage terminal capacitance partial voltage around the power line, and obtained 16.4 mW energy under the voltage of 60 kV through laboratory and field tests [43,44]. R. Moghe et al. proposed anon-cylindrical structure with plate electrodes for harvester electric field energy and built a medium-voltage prototype integrated with a voltage sensor. Experimental results showed a continuous power of 17 mW at 35 kV bus voltage [45,46]. Similar work has also been reported in the literature [47].

### 4.4. Low-Potential EEH

The principle of low-potential EEH is quite similar to the high-potential EEH method, except that the whole load locates at the low potential side; see Figure 3. The low-potential capacitor (electrodes) are both with suspended potential and have no direct connection with the HV conductor. As the size of spatial electrodes is limited, the impedance of spatial capacitance *C*_0_ is usually much greater than that of the load. Therefore, *C*_0_ shares the majority of the full operating voltage of the HV conductor, and the EEH module only withstands lower voltage. Consequently, a strict insulation structure is unnecessary in a low-potential EEH system. However, the disadvantage is that it can only provide a small amount of the scavenged power for the load, and there may be difficulties to support or fix the spatial electrodes while in its field installation.

J. Rodríguez et al. proposed a novel approach for optimum electric-field energy harvesting using the parasitic capacitance of medium-voltage power line insulators, and studies show the potential to obtain nearly 100 mW from a 12.7 kV power line [48,49]. S. Kang et al. used a conductor tube under actual three-phase 765 kV power transmission lines to harvest electric-field energy and successfully power a Zigbee-based temperature sensor node [50]. Except for the applications on transmission lines, E. B. Pehlivanoglu et al. used a metal panel against a transformer to harvest electric field energy and it is investigated with a transformer room experimental set-up. The results demonstrate that 40 mJ of energy can be harvested in a period of 900 sec [51]. O. Cetinkaya et al. proposed a novel power provision architecture by exploiting E-fields emitted by lighting elements to IoT-enabled wireless commutations [52].

The application of EEH from an HV conductor is technically worthwhile, as the magnitude of E-field energy is proportional to the square of the applied voltage. However, for applications such as IoT sensors orientating low-voltage appliances, EEH from low-voltage power lines may be challenging. M. Honda et al. adopted electrodes attached on the insulating cover of two-wire power cords and 3–4 V, 1.4µW is harvested with 20 cm electrodes from a 100 V AC power supply [53]. I.B.Vendik placed two multilayer electrodes over one cable without a direct connection to the ground and 600 mV output voltage was obtained [54]. A stick-on capacitive energy harvester that harvests the stray electric field generated around AC power lines without a reference connection to earth ground and harvest 270.6 µJ of energy from a 14 cm long interface in 12 min [55]. Similar work has also been reported in the literature [56,57,58,59].

To further improve the efficiency of EEH, the high performance of subsequent energy management system is another critical enabling technology. O. Menéndez studied a mechatronized maximum power point tracking system to automatically vary the location of the electrodes for harvesting the maximum electric-field power. The results show that the harvested power rises by approximately 94%, with a power density of 0.04l W/cm^2^ in non-contact applications [59]. J. Rodríguez proposed flyback conversion in pulsed energy transfer mode so that the system is self-triggered to harvest 23.6 mW from a 12.7 kV power line [60]. P. Li et al. proposed a low-power-consumption high-efficiency up-conversion MPPT matching management circuit for the electric field energy harvesting, which can continuously accumulate weak energy from the power-line transducer for a long period and provide higher power output in a very short time [61].

To sum up, the investigations of EEH covers geometry optimization of the capacitor structure and strategies for energy management. Besides, the impact of the complex external electric field in real-life environments, such as substation also needs to be considered to design the harvester. However, existing works are mostly conducted under laboratory simulated uniform E-field, and the impact on the charge collection needs to be further investigated in real-life E-field. Another critical issue is that the collected electric energy is limited, and the complexity of the subsequent voltage condition module can be energy-intensive, which directly compromise the total efficiency of an EEH system. Therefore, in order to satisfy the requirement of operating voltage and power consumption of most sensors, it is necessary to further investigate the simple, efficient and low-power voltage conditioning and storage technology.

## 5. Conclusions

This paper provides a review of the investigations on electric field energy and magnetic field energy harvesting for achieving energy-autonomous condition monitoring sensors or devices in the power grid scenario. Firstly, this paper analyzes the potential environmental energy in the power system and shows that the magnetic field and electric field are the two most available energies in this scenario. Then, E-field and H-field energy harvesting are reviewed, respectively, based on the classifications of different harvesters’ configurations. Among the several energy harvesting methods reviewed in this paper, high-potential MEH has the highest output power but with limited application scope, which is only suitable for energy consumptive applications in the vicinity of transmission lines such as video monitoring of the lines. The produced power of low-potential MEH and EEH is lower, but with wider serviceable range, which can be used in low-power sensing of some simple parameters in the grid.

For high-potential MEH energy harvesting, future research mainly focuses on the strategies to ensure the harvested energy suffices for the loading power consumption under small current in HV lines, while preventing the core from saturation under large current and protecting the system from overvoltage generated from short-circuit fault current. For low-potential MEH and EEH, it is necessary to further investigate the simple, efficient and low-power voltage conditioning and storage technology.
